# Microfluidic single-cell transcriptional analysis rationally identifies novel surface marker profiles to enhance cell-based therapies

**DOI:** 10.1038/ncomms11945

**Published:** 2016-06-21

**Authors:** Robert C. Rennert, Michael Januszyk, Michael Sorkin, Melanie Rodrigues, Zeshaan N. Maan, Dominik Duscher, Alexander J. Whittam, Revanth Kosaraju, Michael T. Chung, Kevin Paik, Alexander Y. Li, Michael Findlay, Jason P. Glotzbach, Atul J. Butte, Geoffrey C. Gurtner

**Affiliations:** 1 Department of Surgery, Hagey Laboratory for Pediatric Regenerative Medicine, Stanford University School of Medicine, Stanford, California 94305, USA; 2 Program in Biomedical Informatics, Stanford University School of Medicine, Stanford, California 94305, USA; 3 Section of Plastic, Aesthetic and Reconstructive Surgery, Johannes Kepler University, Linz, Austria; 4 Department of Plastic Surgery and Hand Surgery, Technical University Munich, Munich, Germany; 5 The University of Melbourne Department of Surgery, Royal Melbourne Hospital, 300 Grattan St Parkville VIC 3068, Australia; 6 Department of Pediatrics, Division of Systems Medicine, Stanford University School of Medicine, Stanford, California 94305, USA

## Abstract

Current progenitor cell therapies have only modest efficacy, which has limited their clinical adoption. This may be the result of a cellular heterogeneity that decreases the number of functional progenitors delivered to diseased tissue, and prevents correction of underlying pathologic cell population disruptions. Here, we develop a high-resolution method of identifying phenotypically distinct progenitor cell subpopulations via single-cell transcriptional analysis and advanced bioinformatics. When combined with high-throughput cell surface marker screening, this approach facilitates the rational selection of surface markers for prospective isolation of cell subpopulations with desired transcriptional profiles. We establish the usefulness of this platform in costly and highly morbid diabetic wounds by identifying a subpopulation of progenitor cells that is dysfunctional in the diabetic state, and normalizes diabetic wound healing rates following allogeneic application. We believe this work presents a logical framework for the development of targeted cell therapies that can be customized to any clinical application.

## Abstract (web summary)

Unrecognized progenitor cell perturbations underlying a disease state may limit the efficacy of cell therapies. Here, the authors use high-throughput, single-cell transcriptional analysis to identify disease-specific cellular alterations and prospectively isolate restorative cell subpopulations.

## Graphical abstract

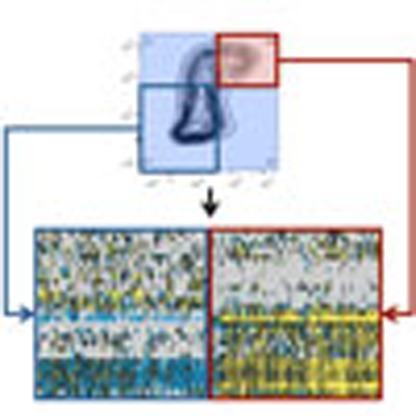

Cell-based therapies have been proposed for regenerative medicine and wound healing applications^1^. Progenitor cell therapies are being tested in clinical trials to either directly address diabetic pathophysiology^2^, or to treat diabetic complications such as retinopathy, critical limb ischaemic and diabetic foot ulcers^3^. However, existing cell-based approaches have been developed primarily empirically based on the ‘legacy’ surface markers (SMs) that were originally described for other cell types^4^, making it difficult to decide how to proceed when trials fail. Recently, there has been an increased understanding of the heterogeneity of stem and progenitor cell populations^5^,^6^, as well as a shift in the mechanistic hypothesis of cell therapies from direct tissue engraftment to enhancement of dysfunctional endogenous repair pathways^7^. Thus, there is a need to rationally develop targeted cell-based approaches for specific clinical applications through the selection of cell subpopulations with desired transcriptional profiles.

Customized cell therapies require an in depth knowledge of both disrupted cellular pathways in diseased tissue and therapeutic cell SM profiles to isolate discrete cell pools for application. Progress has been made in understanding gross repair pathway disruptions in diseased tissues, which provides a basis for rationally replacing deficient growth factors and cytokines^8^,^9^,^10^,^11^. While enrichment of progenitor cells has shown therapeutic promise^12^,^13^, a more granular understanding of the subpopulation dynamics of diseased and therapeutic progenitor cell pools has proven challenging because the resolution afforded by traditional population-level assays is insufficient to capture the complex relationships in heterogeneous cell populations^14^,^15^,^16^. Standard approaches rely on pooling RNA or protein from hundreds of thousands of cells to report aggregate gene expression, and are thus unable to detect differential distributions in gene expression among cell subgroups. Recent advances in high-throughput, microfluidic technology have enabled massively parallel single-cell gene expression analyses, with the resulting data providing insights into the relationships among cells in complex tissues^17^,^18^,^19^,^20^. Leveraging this technique in previous work, we have combined single-cell transcriptional analysis with advanced mathematical modelling to characterize heterogeneity in putatively homogeneous populations, as well as identify critical perturbations in cell subpopulations in pathologic states^21^,^22^,^23^,^24^. Most recently, we have utilized single-cell analysis to link defects in the neovascular potential of diabetic and aged progenitor cells to the selective depletion of specific cell subsets^25^,^26^,^27^. These findings support the concept of functional heterogeneity within progenitor cell pools and highlight the potential of highly selected cell therapies to reverse specific cellular and pathophysiologic defects in diabetic and other impaired tissues.

In this work, we sought to create a rational framework to develop targeted cell therapies from heterogeneous progenitor populations for specific clinical diseases such as diabetes. Specifically, we hypothesized that single-cell transcriptional analyses could prospectively identify physiologically distinct progenitor cell subpopulations depleted in diabetes and with enhanced wound healing activity, based on the differences in individual cell gene expression distributions. Furthermore, the parallel assessment of intra-cellular and surface targets would enable subpopulation enrichment for therapeutic application by providing novel cell surface ‘recipes’. Importantly, this approach was designed to identify subpopulation-defining SMs comprehensively (by testing all 386 markers with commercially available antibodies) and blindly (assuming no mechanistic hypothesis). This comprehensive, blind approach greatly expands the potential SM pool and increases the likelihood of identifying subpopulations with robustly expressed markers to select cells.

## Results

### Stem cell subpopulation and SM identification

**Figure 1:**
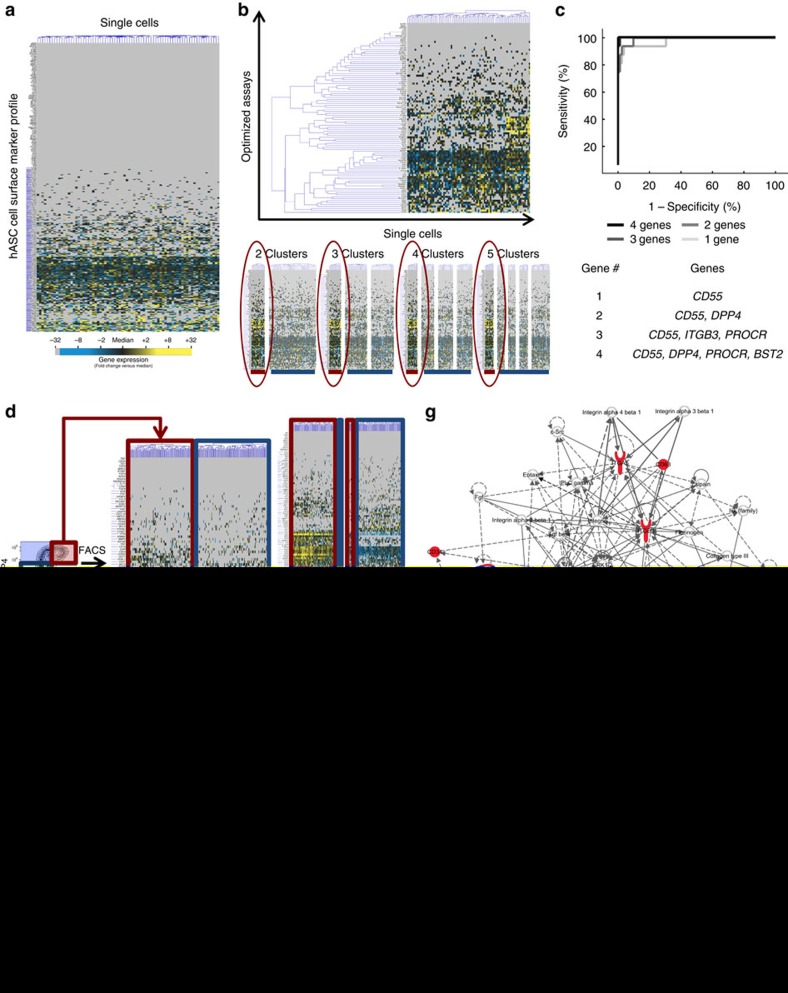
Single-cell transcriptional analysis identifies a subpopulation of human ASCs with putatively enhanced regenerative potential. (**a**) Single-cell transcriptional screening of all known cell SMs to identify those with differential expression (most useful for cell subtyping). Gene expression presented as fold change from median (yellow—high expression, 32-fold above median to blue—low expression, 32-fold below median; grey—no expression). (**b**) Single-cell analysis focused on high copy number, differentially distributed SM genes identified a cell subpopulation present across repeated k-means clusterings. (**c**) Linear discriminate analysis (LDA) identified SMs for prospective subpopulation isolation, with ROC analysis of cluster sensitivity and specificity utilizing the ‘best’ individual or groups of genes determined using forward feature selection. (**d**) Single-cell confirmation of prospective hASC subpopulation isolation via FACS using two LDA-defined SMs (DPP4 and CD55). (**e**) Positive hASC subpopulation enrichment enhances gene expression distributions for multiple genes related to tissue regeneration (selected significantly affected genes displayed as determined via Kolmogorov–Smirnov testing). (**f**) Single-cell whisker plots and pooled cell RT-PCR demonstrating a confirmation of selected single-cell gene distribution findings on a population level. (**g**) Top scoring IPA-constructed transcriptome network based on the genes significantly increased following positive hASC selection. Significant ‘seed’ genes are coloured in red to distinguish them from the remaining ‘inferred’ entities in the network. *indicates *P*≤0.05 for positive selection versus hASCs or negative selection, via one-way ANOVA. Error bars represent s.e.m.

Utilizing human adipose-derived stem cells (hASCs) as a test progenitor cell pool, we first obtained a comprehensive profile of hASC SM expression through single-cell transcriptional analysis of ‘all’ known SMs with commercially available antibodies (Fig. 1a, Supplementary Data 1). This allowed us to cast the widest possible net in our search for novel subpopulation-defining markers without relying on *a priori* assumptions of gene expression. Using this approach, we identified over 200 markers that were expressed within hASCs. Focusing on the ∼90 SMs with highest, non-uniform expression (which are most likely to distinguish biologically important cell subsets; Supplementary Fig. 1), we identified a distinct subpopulation of hASCs that was consistently present across multiple partitional clustering permutations (Fig. 1b). This subpopulation could be defined with high sensitivity and specificity by two SM genes (*DPP4* and *CD55*; Fig. 1c) and was present across multiple human patients (Supplementary Fig. 2a–d). To confirm that this was not an artefact, this transcriptionally defined subpopulation was recapitulated on a protein level (Supplementary Fig. 2a–b), and could be prospectively isolated based on the protein co-expression of DPP4 and CD55 using fluorescence-activated cell sorting (FACS) (Fig. 1d). To predict the function of this subpopulation, we first identified the targets overexpressed in our initial experiment (Supplementary Table 1) and evaluated their function based on the published literature. We found that these cells expressed increased levels of general stem cell markers (such as CD34 and CD73), as well as genes associated with cancer stem cells (CD99 and ITGB3) and embryonic stem cells (GGT1), suggesting that this subpopulation may have increased regenerative and wound healing potential^28^,^29^,^30^.

### Stem cell subpopulation verification and *in vitro* characterization

To confirm that these cells were functionally distinct from the parent pool, we performed repeat single-cell transcriptional analyses with a new gene list focused on intra-cellular targets thought to be important in wound healing (Supplementary Fig. 2e)^8^,^9^,^10^,^11^. We found that the identified cell subset, defined by and enriched via DPP4 and CD55 expression, displayed a transcriptional profile with increased expression of ‘intra-cellular’ genes related to cell survival, stemness, blood vessel growth and tissue remodelling (Fig. 1e–f, Supplementary Figs 2f, 4b). To extend our results beyond the 96 genes explicitly analysed, canonical pathways overrepresented in these cells were determined using ingenuity pathway analysis (IPA) (Fig. 1g). These inferred pathways included multiple cardiovascular and wound healing processes, lending additional support to an enhanced wound healing potential for this discrete cell subset.

**Figure 2:**
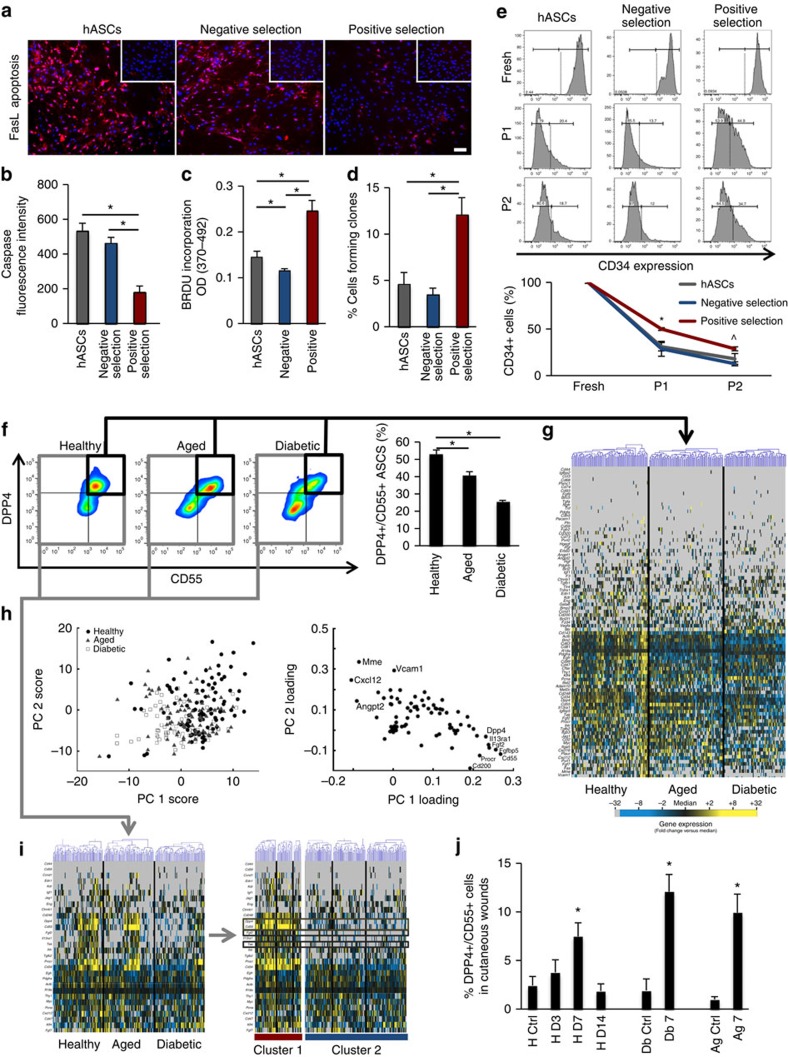
Effect of prospective hASC selection and co-morbidities on ASC subpopulation dynamics to inform cell source decisions. (**a**,**b**) Enrichment for the transcriptionally identified hASC subpopulation enhances cell survival following exposure to an *in vitro* apoptotic stimulus (Fas ligand; measuring caspase activation (red)), (**c**,**d**) increases cell proliferation and clonogenecity and (**e**) prolongs stemness marker (CD34) expression. (**f**,**g**) The transcriptionally identified ASC subpopulation is significantly depleted and possesses deregulation of critical signalling pathways visible on single-cell analysis in the setting of both diabetes and aging. Gene expression presented as fold change from median (yellow—high expression, 32-fold above median to blue—low expression, 32-fold below median; grey—no expression). (**h**) Principal component projections of individual cells (left) and genes (right) demonstrating considerable segregation among phenotypes, driven largely by vascular/tissue remodelling genes. (**i**) Single-cell transcriptional analysis of healthy, aged and diabetic mASCs reveals that the depletion/dysfunction of cluster 1 cells in these states is not a the result of cell SM loss and redistribution to other clusters (expression profiles of subpopulation-defining SMs and tissue remodelling genes highlighted). (**j**) Flow cytometric analysis demonstrating dynamic DPP4/CD55 subpopulation increases in wild-type wounds, supporting their role in the wound healing process. The DPP4/CD55 subpopulation was also elevated in diabetic and aged wounds as compared with uninjured skin, with a trend toward compensatory overrecruitment consistent with an impaired cellular functionality. *indicates *P*≤0.05 via one-way ANOVA or Student’s *t*-test (healthy versus aged or diabetic in **f**; day 7 versus respective controls in **j**). ^∧^indicates *P*≤0.05 for positive versus negative selection via Student’s *t*-test. Error bars represent s.e.m. Scale bar, 50 μm.

We next sought to confirm the functional importance of the DPP4/CD55 subpopulation. Starting *in vitro* we assessed cell survival, stemness, proliferation and colony-forming capacity, as these are idealized characteristics of effective cell therapies. As predicted, subpopulation enrichment led to enhancement in cell survival following exposure to an apoptotic stimulus (Fig. 2a,b, Supplementary Fig. 3), improved cell robustness as determined by proliferation and colony-forming capacity (Fig. 2c,d), and prolonged expression of cell stemness markers across extended passages (Fig. 2e).

### Disease effects on stem cell subpopulation dynamics

We have previously catalogued the depletion of progenitor cell subsets from therapeutic cell populations in murine disease models^25^,^26^,^27^, consistent with the hypothesis that cellular perturbations underlie disease-specific sequelae. Building on this work, we verified that this DPP4/CD55 subpopulation was abundantly present in the adipose tissue of healthy mice (Supplementary Fig. 4). However, we found that both diabetes and aging were associated with decreases in the numbers of these cells in adipose tissue (Fig. 2f), as well as deregulation of critical cellular wound healing signalling pathways (Fig. 2g,h). This cellular dysfunction was not explained by SM drift (Fig. 2i), and was confirmed in humans in the setting of diabetes (Supplementary Fig. 5a). These data support the disruption of a highly functional subset of progenitor cells as a global mechanism for disease-specific complications and impaired therapeutic cell functionality.

Consistent with the critical role of adipocyte precursors in physiologic wound healing^31^, and supporting the clinical potential of the identified cell subset, we found increased levels of DPP4/CD55 cells in the healing wounds of healthy mice, which returned to basal levels after wound closure (Fig. 2j). Increases in the DPP4/CD55 subpopulation were also seen in diabetic and aged wounds (Fig. 2j), however, trends toward compensatory overrecruitment were observed in these pathologic states, consistent with an impaired or deleterious cellular functionality. These data support a diminished therapeutic potential of autologous cells in these pathologic settings, even following subpopulation enrichment, and suggest that allogeneic cell pools may be more beneficial in patients with underlying disease.

### Therapeutic efficacy of stem cell subpopulation enrichment

**Figure 3:**
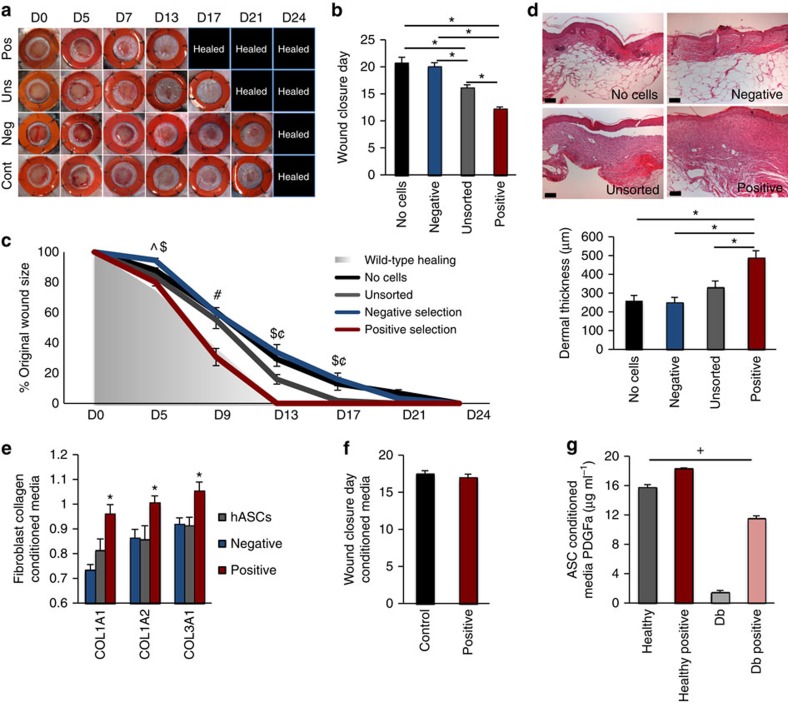
Prospective ASC selection enhances *in vivo* wound healing potential. (**a**–**c**) A single, local application of enriched mASCs to murine diabetic wounds significantly accelerates wound closure rates (tracked via digital photography and serial wound area measurements) as compared with unsorted or negatively selected cells, essentially normalizing diabetic murine wound healing kinetics. (**d**) Application of enriched mASCs to diabetic wounds also significantly increases dermal regeneration. (**e**) Supporting a paracrine mechanism of action, enriched ASCs significantly upregulate fibroblast collagen gene expression following exposure to enriched ASC conditioned medium, likely via increased growth factor expression (**g**). (**f**) Conditioned medium from enriched ASCs does not have the same beneficial effect on diabetic wound healing as direct cell application despite enhanced growth factor expression (**g**), highlighting the importance of sustained cytokine secretion with live-cell therapies. (**g**) Enrichment of diabetic ASCs does not correct growth factor deficiencies, supporting the use of allogenic cells. For wound healing curves, *P*≤0.05 via one-way ANOVA indicated by: ^∧^for positive versus negative selection; ^$^for negative selection versus unsorted cells; ^#^for positive versus all groups; ^¢^for positive selection versus no cell control. ^+^indicates *P*<0.05 via one-way ANOVA for all comparisons. *indicates *P*≤0.05 via one-way ANOVA or Student’s *t*-test for remaining data (positive versus negative selection and hASCs in **e**). Error bars represent s.e.m. Scale bar, 100 μm.

Guided by these findings, we evaluated the *in vivo* therapeutic potential of subpopulation-enriched, allogeneic ASC-based therapies in the setting of impaired murine diabetic wounds. As predicted based on the transcriptional and *in vitro* enhancements of the identified ASC subpopulation, and its dysregulation in the diabetic state, a single application of FACS-enriched healthy ASCs accelerated wound closure rates and improved dermal regeneration compared with application of unsorted or depleted cells, effectively ‘normalizing’ diabetic healing to wild-type kinetics (Fig. 3a–d). These cells persisted for up to 16 days *in situ* (Supplementary Fig. 5b), likely acting via IPA-predicted cytokine-mediated improvements in local wound healing pathways, and the upregulation of fibroblast collagen production (Fig. 3e,g)^32^. In the field of wound healing, very few interventions are capable of ‘normalizing’ the wound healing timeline, demonstrating the power of this approach. Importantly, depletion of this ASC subpopulation completely abrogated the beneficial effects of cell therapy (Fig. 3a–e), and enriched medium did not have the same beneficial wound healing effect as a single direct cell subpopulation application (Fig. 3f). Consistent with our single-cell data demonstrating impairment of diabetic ASCs across subpopulations, enrichment of diabetic ASCs did not restore their wound healing capacity (Fig. 3g, Supplementary Fig. 6a). These data suggest that healthy, enriched ASCs are both necessary and sufficient to maximally improve wound healing using this approach.

Regarding the safety of ASC application, one-time dosing was used to limit allogenecity, and cell applications were tolerated without local or systemic signs of rejection. Moreover, treated wounds demonstrated considerable vascularity on closure (Supplementary Fig. 6b), with no evidence of tumour formation or wound breakdown 3 months after application. Further increasing the clinical potential of these findings, rapid subpopulation enrichment of hASCs was also possible via magnetic-assisted cell sorting (MACS) for the cluster-discriminating SMs DPP4 and CD55 (Supplementary Fig. 6c), enabling a total processing time (from tissue collection to cell application) on the scale of an hour. These data demonstrate the potential utility of targeted cell subpopulation enrichment for cell-based therapeutics utilizing a process that is adaptable to virtually any pathologic state.

## Discussion

This work establishes the efficacy of single-cell analysis for the rational enhancement of cell-based therapeutics by addressing pathologic alterations in progenitor cell biology. Importantly, this methodology overcomes the inherent challenges progenitor cell heterogeneity imposes on our understanding of pathologic cellular perturbations and the standardization of cell-based approaches, and enables the logical assessment and development of targeted cell therapies addressing an underlying cause of specific clinical defects. Importantly, by making no *a priori* assumptions of cell SM expression, this approach is adaptable to any cell population. It also definitively informs decisions regarding the utility of autologous versus allogeneic cell sources.

hASCs were the target population assessed here due to their therapeutic potential, limited immunogenecity and safety profile^33^,^34^,^35^,^36^,^37^. Our findings build on our previous work demonstrating ASC subpopulation perturbations in diabetes^27^, critically identifying novel SMs for prospective subpopulation isolation, testing and therapeutic application. While there are currently no FDA-approved progenitor cell treatments for superficial wounds, multiple products containing mesenchymal progenitors are in early stages of clinical testing^1^. Our data support the safety and effectiveness of hASC therapies for the treatment of diabetic wounds, and suggest that sub-fractionation of other progenitor cell populations may similarly enhance their therapeutic potential in this setting. On the basis of these findings, future direct comparisons with other treatment modalities are warranted. This experimental framework did not seek to address the underlying defects of diabetes. We nonetheless envision that a similar methodology would be beneficial to more curative cell therapies, such as pancreatic islet cell transplantation. Given the adaptability of this approach to any cell type, the authors feel this technique has the potential to standardize and improve cell-based therapies for any disease state.

## Methods

### hASC isolation

Human abdominoplasty specimens were obtained after acquiring informed consent from patients, in accordance with the Stanford University Institutional Review Board guidelines. For initial experiments, ASCs were collected from the tissue samples of multiple adult female patients without major medical conditions who were undergoing elective abdominoplasty procedures. For experiments on the effect of diabetes on hASC subpopulations, ASCs were isolated from consecutive patients (*n*=3) undergoing elective bariatric surgery who met predefined criteria for diabetes mellitus (haemoglobin-A1c (HgbA1c)>6.5). Controls for these experiments were adult patients without major medical conditions undergoing elective abdominoplasty (*n*=4) without a history of diabetes mellitus or HgbA1C⩾6.0). Human ASCs from all groups were isolated based on an established protocol^38^. Raw human abdominoplasty specimens were manually minced, washed and treated with 0.075% collagenase type I (Sigma-Aldrich, St Louis, MO) in Hank’s balanced salt solution (Life Technologies, Grand Island, NY) for 1 h at 37 °C with gentle agitation. The reaction was stopped with the addition of fetal bovine serum (FBS), and after centrifugation, the pelleted stromal vascular fraction was prepared for FACS as described below.

### Animals

Young (3 months) and aged (21 months) wild-type mice (C57BL/6), and young diabetic mice (db/db; BKS.Cg-Dock7m^+/+^ Leprdb/J) were obtained from Jackson Laboratories (Bar Harbor, ME) and the National Institute on Aging (NIA, Bethesda, MD). Luciferase^+^/GFP^+^ mice (FVB-Tg(CAG-luc,-GFP)L2G85Chco/J) were also obtained Jackson laboratories. All protocols were approved by the Stanford Administrative Panel on Laboratory Animal Care.

### mASC isolation

Murine ASCs (mASCs) were isolated from young, aged, diabetic and luciferase^+^/gfp^+^ murine inguinal fat pads, minced and digested for 1 h at 37 °C using collagenase I (Sigma-Aldrich). After quenching the reaction and centrifugation, the pelleted stromal vascular fraction (SVF) was prepared for FACS as described below.

### FACS

Fresh human or mASCs were sorted on a FACS Aria II instrument (BD Biosciences, San Jose, CA) with the use of a 100-μm nozzle. Cells were isolated as described above, and incubated for 20 min in FACS buffer (phosphate-buffered saline (PBS) supplemented with 2% FBS) containing one of the following antibody combinations: (1) anti-human ef-450-conjugated CD45 (eBioscience, San Diego, CA), APC- or PE-conjugated CD34 (BD Biosciences), FITC-conjugated CD31 (BD Biosciences), PE- or APC-conjugated DPP4 (BD Biosciences) and PE-Cy7-conjugated CD55 (Biolegend, San Diego, CA); (2) anti-mouse ef-450-conjugated CD45 (eBioscience), APC-conjugated CD34 (Biolegend), PE-Cy7-conjugated CD31 (BD Biosciences), FITC-conjugated DPP4 (BD Biosciences) and PE-conjugated CD55 (Biolegend); or (3) anti-mouse PE-Cy7-conjugated CD45 (Biolegend), APC-conjugated CD34 (Biolegend), ef-450-conjugated CD31 (eBioscience), APC-Cy7-conjugated DPP4 (Abcore, Ramona, CA) and PE-conjugated CD55 (Biolegend). Using a Becton Dickinson flow cytometric cell sorter, cells were either sorted as single cells into 6 μl of lysis buffer for single-cell transcriptional analysis, or as populations for subsequent culture. ASCs were defined with the SM profile CD45^−^/CD31^−^/CD34^+^ (to exclude contaminating hematopoietic and endothelial cells found within the SVF), and CD55 and DPP4 expression within this ASC population was used for positive and negative subpopulation selection (see Supplementary Fig. 2a,b for gating scheme). Cells sorted for culture were plated onto conventional tissue culture plates in the DMEM (Life Technologies) supplemented with 10% FBS and 1% P/S. Plated cells were cultured under standard conditions (37 °C and 5% CO_2_) and used at or before passage two. *In vitro* assays were run in triplicate unless otherwise stated.

### *In vitro* survival assays

hASC survival was assessed using two methods following exposure to the apoptotic stimulus Fas ligand (1, 10 or 100 ng per ml for 5 h; Human Recombinant Super FasL; Enzo Life Sciences, Farmingdale, NY). Apoptotic caspase activation was assessed using the CaspaTag Caspase 3–7 kit (Millipore, Billerica, MA) according to the manufacturer’s instructions. Briefly, cells were seeded on a chamber slide, and following exposure to FasL, cells were washed and incubated for 1 h at 37 °C with the CaspaTag reagent solution. After washing, red fluorescence was captured using a Leica DM5000 microscope (Leica Microsystems, Inc., Wetzlar, Germany) equipped with a DFC300FX camera. Hoescht staining was used to label nuclei, and untreated cells were used a control. Fluorescence intensity (595 nm) was analysed using Image J software (NIH, Bethesda, MD). Brightfield images were also obtained for assessment of apoptotic cell morphology.

Downstream annexin V activation was also assessed following FasL exposure using the Mitochondrial Membrane Potential/Annexin V Apoptosis kit (Invitrogen, Carlsbad, CA), according to the manufacturer’s instructions. Following exposure to the FasL, cells were washed, lifted using trypsin and incubated with MitoTracker Red dye for 30 min at 37 °C and 5% CO_2_. Cells were then washed, incubated in Alexa Flour 488 annexin V for 15 min, and analysed via flow cytometry (FACS Aria II instrument; BD Biosciences). Untreated cells were used as negative controls.

### Cell proliferation

hASC proliferation was assessed following FACS sorting using a BrdU proliferation assay. Sorted hASCs (2 × 10^3^) were seeded in 96-well plates under standard culture conditions (DMEM with 10% FBS, 37 °C and 5% CO_2_). After reaching ∼70% confluence, the cells were serum starved in DMEM containing 0.5% FBS for 24 h to induce quiescence. Fully supplemented medium was then re-applied for 18 h. BrdU (Cell Proliferation ELISA, BrdU; Roche, Basel, Switzerland) was then added to the medium, and after an additional 6 h, cell proliferation was assayed according to the manufacturer’s instructions.

### Colony-forming assay

hASC clonogenic capacity was assessed by FACS sorting single cells into each well of 48-well plates containing standard cell growth medium. Medium was changed every 5–7 days, and at day 14 the number of cells forming colonies (defined as a cluster of 30 cells or greater) was manually counted using standard light microscopy.

### Real-time quantitative PCR

Total RNA was isolated from sorted primary hASCs, diabetic mASCs or human dermal fibroblasts (Life Technologies) using the RNeasy Mini Kit (Qiagen, Germantown, MD) and transcribed to cDNA (Superscript First-Strand Synthesis Kit, Invitrogen). Real-time qPCR reactions were performed using Taqman gene expression assays (Applied Biosystems, Foster City, CA) for human *DPP4* (dipeptidyl-peptidase 4, Hs00175210_m1), *CD55* (CD55 molecule, decay accelerating factor for complement, Hs00892618_m1), *TEK* (Endothelial Tyrosine Kinase, Hs00945146_m1), *CD248* (CD248 molecule, endosialin, Hs00535586_s1), *JAG1* (jagged 1, Hs01070036_m1), *CD47* (CD47 molecule, Hs00179953_m1), *COL1A1* (Collagen, Type I, Alpha 1, Hs00164004_m1), *COL1A2* (Collagen, Type I, Alpha 2, Hs01028956_m1) or *COL3A1* (Collagen, Type III, Alpha 1, Hs00943809_m1); or murine *Col1a1* (Mm00801666_g1), *Col1a2* (Mm00483888_m1) or *Col3a1* (Mm01254476_m1) using a Prism 7900HT Sequence Detection System (Applied Biosystems). Expression levels of the target genes were normalized to *R18S* (Eukaryotic 18S rRNA, Hs99999901_s1), *GAPDH* (Glyceraldehyde 3-phosphate dehydrogenase, Hs99999905_m1) or *Actb* (β-actin, Mm01205647_g1). Relative gene expression across conditions was calculated following intra-donor normalization.

### Murine wound healing model

Four-month-old male diabetic mice (db/db; BKS.Cg-Dock7m^+/+^ Leprdb/J, Jackson Laboratories) were randomized into four treatment groups: no cells, unsorted cells (SVF) and positively selected (DPP4^+^/CD55^+^) and negatively selected (DPP4^−^/CD55^−^) cells, isolated from healthy, male 4-month-old wild-type mice via FACS as described above (*n*=10 wounds per condition). Using an established protocol^39^, two 6 mm full-thickness cutaneous wounds were excised on either side of the midline of the murine dorsum, with each wound stented with silicone rings sutured in place to prevent wound contraction. Following wounding, a total of 5 × 10^5^ cells in 80 μl of saline was injected sub-dermally in four sites around the wound edge, with the no cell control group receiving saline injections only. All wounds were covered with an occlusive dressing (Tegaderm, 3M, St Paul, MN). Digital photographs were taken on day 0, 5, 9, 13, 17, 21 and 24. Wound area was measured using Image J software (NIH). On closure, wounds were collected and fixed in 4% paraformaldehyde overnight and embedded in paraffin, or immediately embedded in OCT (Sakura Finetek USA, Inc., Torrance, CA). For analysis of dermal thickness, paraffin sections were stained with hematoxylin and eosin (H&E, Sigma-Aldrich) and average thickness was calculated from three measurements per high-power field per wound. To assess vasculature in healed wounds, 7-micron thick frozen sections were immunohistochemically stained for CD31 (1°—1:100 Rb α CD31 (Ab28364; Abcam, Cambridge, MA); 2°—1:400 AF547 Gt α Rb, Life Technologies), with nuclei stained with DAPI.

The effect of DPP4/CD55 ASC conditioned medium on diabetic wounds was assessed using the same wound model in diabetic mice. Excisional wounds were created as above, and treated at day 0 with 200 μl of conditioned medium collected from primary sorted DPP4/CD55 mASCs (48 h incubation with DMEM/0.5% FBS under standard culture conditions), or PBS, using a pullulan-collagen hydrogel biodegradable scaffold^40^. Wounds were tracked until closure using digital photographs as above.

The murine wound healing model was also used to assess DPP4^+^/CD55^+^ cell presence in healthy, diabetic and aged wounds, as compared with uninjured skin (*n*=4 wounds per condition). Briefly, cutaneous wounds were created on young (3 months) and aged (21 months) wild-type mice (C57BL/6, Jackson Laboratories), and young diabetic mice (db/db; BKS.Cg-Dock7m^+/+^ Leprdb/J, NIA). Cutaneous wounds were collected at days 3, 7 and 14, and the tissue was minced and digested for 1 h at 37 °C using collagenase I (Sigma-Aldrich). Uninjured skin was used as controls for each group. After quenching the reaction and centrifugation, the pelleted SVF was prepared for FACS as described above. To exclude hematopoietic lineage cells from this analysis, DPP4/CD55 cells were reported as the percentage of CD45^−^ cells that were CD31^−^/CD34^+^/DPP4^+^/CD55^+^.

### *In vivo* bioluminescence imaging

Viability of mASCs was assessed *in vivo* in diabetic mice using bioluminescence imaging (*n*=6 wounds per condition) based on an established protocol^40^. Wounded mice were treated with 2.5 × 10^5^ luciferase positive, DPP4/CD55 enriched or unsorted ASCs injected circumferentially in the wound bed, as above. Mice were anaesthetised at multiple time points and injected with 150 mg kg^−1^ luciferin in PBS intraperitoneally. Images were obtained 10 min later using a 30 s exposure time with a cooled CCD camera using the Xenogen IVIS 200 System (Caliper Life Sciences, Mountain View, CA). Luminescence was quantified as units of total flux in an area of interest subtracted from the background luminescence. Images were taken serially until no bioluminescence was detected.

### ASC modulation of fibroblast collagen production

The effect of DPP4/CD55 healthy and diabetic ASCs on fibroblast collagen production was assessed following fibroblast exposure to ASC conditioned medium. Conditioned medium was collected from primary sorted DPP4/CD55 positive, negative and control hASCs, or positive and control mASCs (24–48 h incubation with DMEM/0.5–1% FBS under standard culture conditions). Human dermal fibroblasts (Life Technologies) seeded at 1 × 10^5^ cells per well in a 6-well plate with DMEM/10% FBS for 24 h, or primarily isolated murine dermal fibroblasts (obtained via overnight incubation in trypsin (Invitrogen) followed by 1 mg ml^−1^ Liberase TL (Roche))^41^, were then exposed to hASC or mASC conditioned medium, respectively, for 48 h. The cells were then washed, and total fibroblast RNA was isolated for RT-PCR as above.

### *In vitro* ASC growth factor release

mASC platelet-derived growth factor-α (PDGF-α) release was assessed following FACS sorting of healthy and diabetic mASCs. Sorted mASCs (2 × 10^4^) were seeded in 24-well plates under standard culture conditions (DMEM with 10% FBS, 37 °C and 5% CO_2_). After reaching ∼90% confluence, the media was switched to DMEM containing 1% FBS. After 24 h the media was collected and protein levels of PDGF-α were quantified using a murine ELISA kit (R&D Systems, Minneapolis, MN) according to the manufacturer’s instructions.

### Magnetic-assisted cell sorting

MACS sorting of hASCs was performed using a MultiSort Kit (Miltenyi Biotec, San Diego, CA). Freshly isolated human SVF was stained with Biotin-conjugated anti-human CD55 (Biolegend) for 20 min on ice, washed, stained with anti-Biotin MicroBeads, and enriched/depleted for CD55^+^ cells using a MACS Separation Column (Miltenyi Biotec). The Multi-Sort Release Reagent was used to dissociate MicroBeads from the cells, which were then labelled with PE-conjugated anti-human DPP4 (Miltenyi Biotec). Cells were washed, stained with anti-PE MicroBeads and enriched/depleted for DPP4^+^ cells using a MACS Separation Column (Miltenyi Biotec). Sorted cells and unsorted controls were stained with FACS antibodies as above for subsequent analysis.

### Microfluidic single-cell analysis

Single-cell reverse transcription and low cycle pre-amplification were performed using an established protocol^22^. Cell suspensions of freshly isolated human or mouse SVF were sorted as single cells into each well of a 96-well plate using a Becton Dickinson FACS Aria flow cytometer into 6 μl of lysis buffer and SUPERase-In RNAse inhibitor (Applied Biosystems). Live/dead gating was performed based on the propidium iodide exclusion. Reverse transcription and low cycle pre-amplification was performed following addition of Superscript III reverse transcriptase enzyme (Invitrogen), Cells Direct reaction mix (Invitrogen), and target gene-specific TaqMan assay (primer/probe) sets (Applied Biosystems; Supplementary Data 1, Supplementary Tables 2–4; 20 min at 50 °C, 2 min at 95 °C, followed by a gene target-specific 22-cycle pre-amplification (denature at 95 °C for 15 min, anneal at 60 °C for 4 min, each cycle)). Exon-spanning primers were used where possible to avoid amplification of genomic background. Sample loading agent (Fluidigm, South San Francisco, CA) and Universal PCR Master Mix (Applied Biosystems) was mixed with the resultant single-cell cDNA and loaded into 96.96 Dynamic Array chips (Fluidigm) along with TaqMan assays (Supplementary Data 1, Supplementary Tables 2–4) and assay loading agent according to the manufacturer’s instructions (Fluidigm). Products were analysed on the BioMark reader system (Fluidigm) using a hot start protocol to minimize primer-dimer formation, 40 quantitative PCR cycles were performed.

### Gene list generation

The approach to selection of ∼90 SM genes for initial subpopulation analysis was one of exclusion, with a focus on SMs with the highest potential for protein based sub-fractionation. Specifically, roughly 400 candidate gene targets with known commercially available antibodies were initially screened via single-cell analysis (Supplementary Data 1). Increasingly restrictive filters were then used to remove targets that would be unlikely to yield FACS-separable subpopulations (Supplementary Fig. 1)^42^. The approach excluded genes not found to be expressed in our target population, followed by those expressed in >95% or <5% of these cells. Of the remaining genes, those with the lowest median cycle threshold value (corresponding to highest median copy number) were included in the initial SM gene list (Supplementary Table 2, Supplementary Fig. 7a). Subsequent human and murine gene lists were then generated using cluster defining genes (Fig. 1b) and intra-cellular targets thought to be important in wound healing (Supplementary Figs 2e,7b,c; Supplementary Tables 3,4).

### Statistical analysis

Results are presented as mean±s.e.m. Standard data analysis was performed using a Student’s *t*-test or one-way analysis of variance (ANOVA), with subsequent comparisons between individual methods completed using a Tukey’s *post hoc* analysis. Wound healing curves were assessed at each time point using one-way ANOVA (historical wild-type murine wound healing curve overlaid for visual comparison). Results were considered significant for *P*≤0.05.

Analysis of single-cell data was performed using an established protocol^21^,^22^. Expression data from all chips for each experiment were normalized to the median expression of each gene in the pooled sample, before being converted to base 2 logarithms. Absolute boundaries defined as ±5 cycle thresholds from the median (or 32-fold increases/decreases in expression) were placed and non-expressing data points were allocated to this floor. To aid data visualization, colour-coded clustergrams were then produced using hierarchical clustering, with a ‘complete’ linkage function and Euclidean distance metric (MATLAB, R2011b, MathWorks, Natick, MA).

To identify subpopulations within this single-cell transcriptional data, k-means clustering was used with a standard Euclidean distance metric, with each cell assigned membership to each cluster as dictated by similarities in gene expression distributions (decreasing the within-cluster sum of square distances in the 96-dimensional gene hyperspace) in MATLAB, and clustering was repeated for k=2 through 5. Optimally partitioned clusters were then sub-grouped using hierarchical clustering to facilitate visualization of data patterning within and across these clusters^22^.

Two-sample Kolmogorov–Smirnov tests were used to identify genes with significantly different distribution patterns between population clusters and/or groups, using a cutoff value of *P*<0.05 with Bonferroni correction for multiple samples. For comparisons among subgroups, the empirical distribution of cells from any given cluster was evaluated against the distribution of all the remaining cells in the experiment.

To identify those SM gene combinations best able to distinguish each cluster, forward feature selection was used using linear discriminate analysis with fivefold cross-validation. The resulting receiver operating characteristic curves were constructed to compare the sensitivity and specificity of each gene set in discriminating the cluster of interest and areas under the curve (AUC) calculated. Selection of the *n*=2 gene model (*CD55* and *DPP4*) was made subjectively based on the breakpoints in the distribution of associated AUC values.

To construct transcriptome networks based on the genes significantly increased following positive selection for the SM proteins CD55 and DPP4, IPA (Ingenuity Systems, Redwood City, CA) was used. In this analysis, the 96 genes from the corresponding single-cell analysis (as opposed to the entire transcriptome) were defined as the reference set (that is, possibility space). This approach was taken to prevent bias of enrichment calculations in IPA’s internal algorithm.

### Data availability

The data that support the findings of this study are available from the corresponding author on request.

## Additional information

**How to cite this article**: Rennert, R. C. *et al*. Microfluidic single-cell transcriptional analysis rationally identifies novel surface marker profiles to enhance cell-based therapies. *Nat. Commun.* 7:11945 doi: 10.1038/ncomms11945 (2016).

## Supplementary Material

Supplementary Information Supplementary Figures 1-7, Supplementary Tables 1-4

Supplementary Data 1 List of 386 surface markers screened via single cell analysis, with percent (%) of total hASCs expressing the gene and the median Ct value.
