# Supplementary material for: Microfluidic single-cell transcriptional analysis rationally identifies novel surface marker profiles to enhance cell-based therapies
Source: Nat Commun. 2016 Jun 21;7:11945. doi: 10.1038/ncomms11945 (PMC5512622; doi:10.1038/ncomms11945)
Supplement: Supplementary Information — Supplementary Figures 1-7, Supplementary Tables 1-4 [file ncomms11945-s1.pdf]

Supplementary Figure 1:

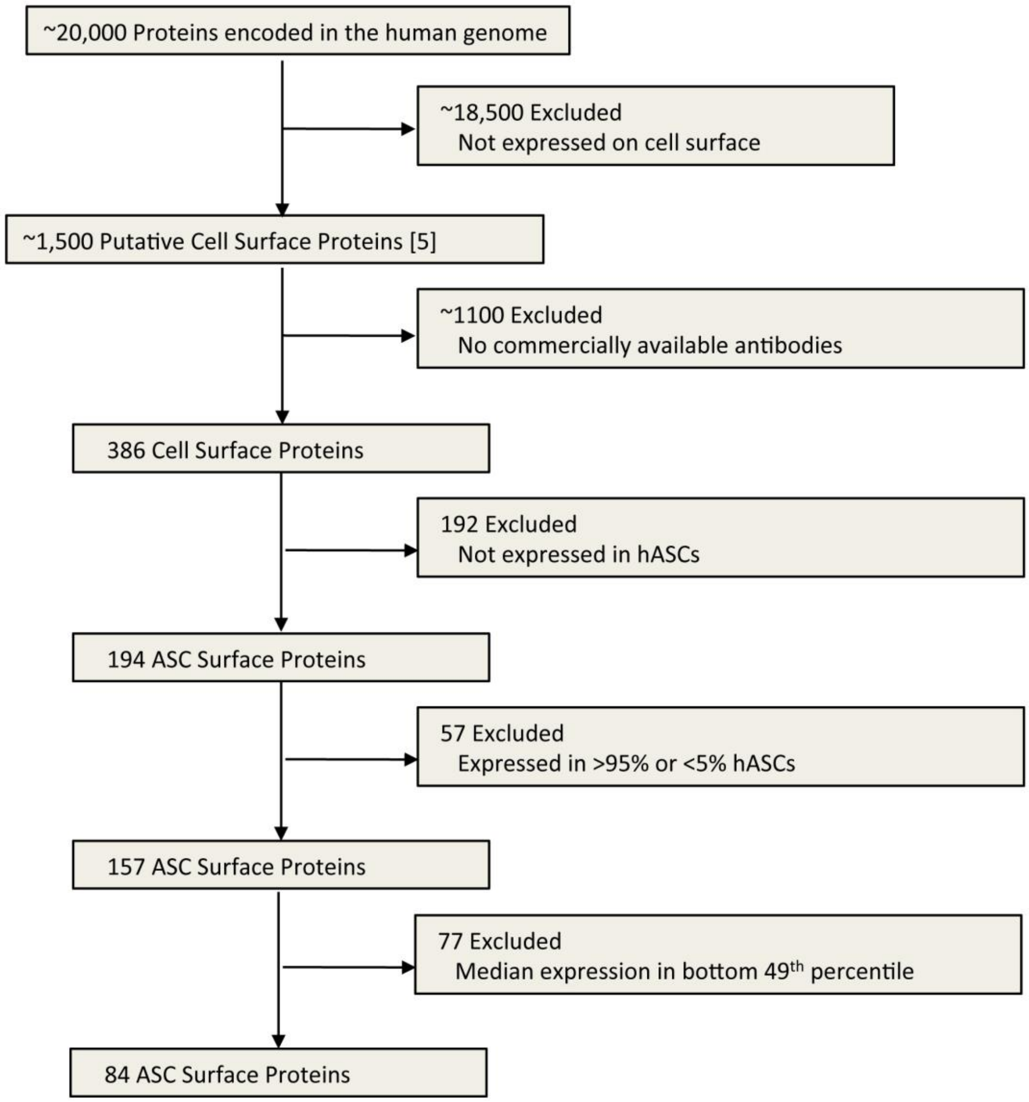

Supplementary Figure 1: Summary of the exclusionary approach to surface marker selection for initial single-cell analysis, with a pragmatic focus on surface markers with the highest potential for protein based sub-fractionation.

Supplementary Figure 2:

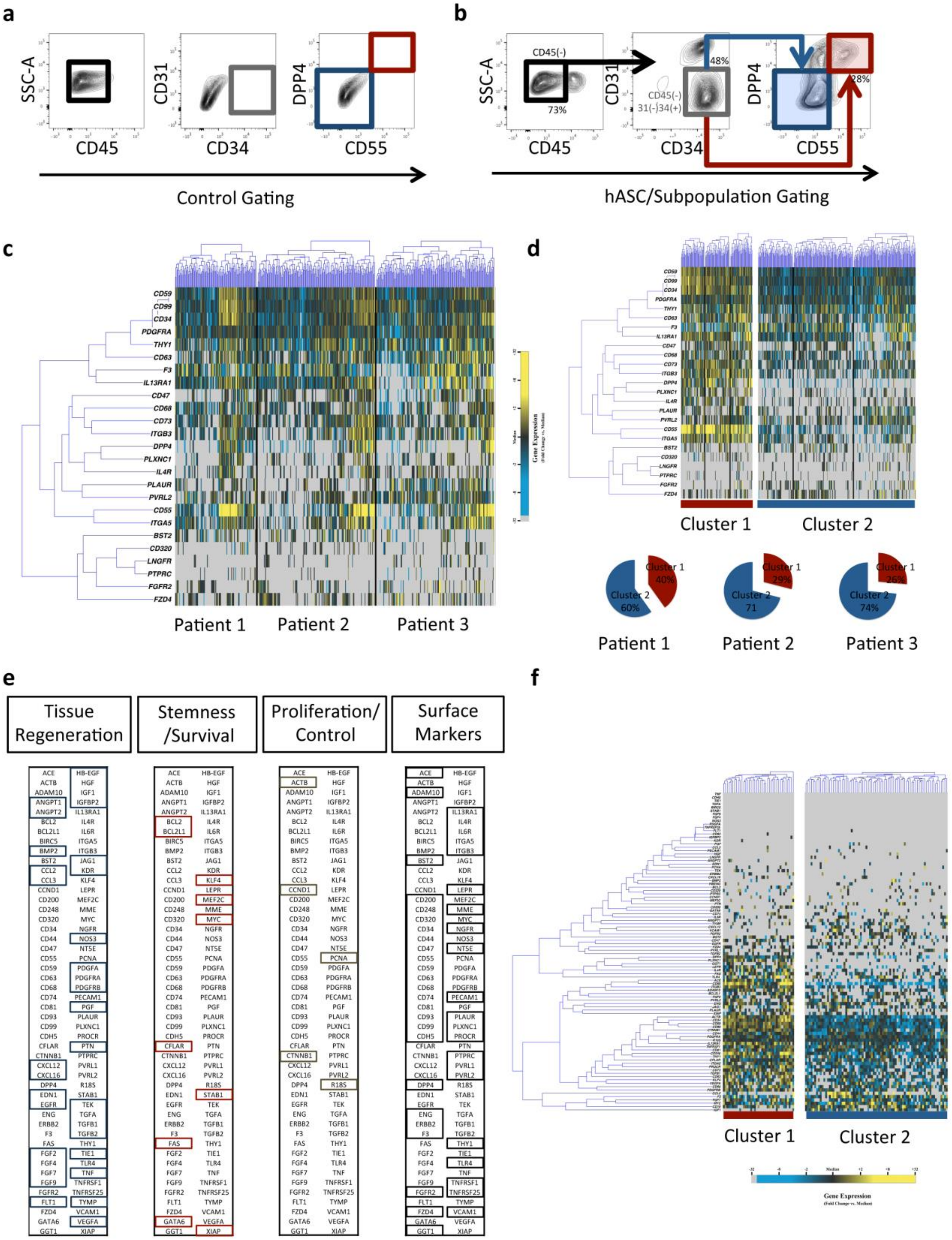

Supplementary Figure 2: hASC subpopulation identification across patient samples and functionalized gene lists. (a-b) Flow cytometry gating scheme for hASCs and subpopulation isolation from the stromal vascular fraction. hASCs were defined as CD45-/CD31-/CD34+ cells within the stromal vascular fraction to exclude contaminating hematopoietic and endothelial cells. DPP4 and CD55 were surface markers used to prospectively isolate the transcriptionally identified hASC subpopulation (%'s reported represent fraction of cells from the parent gate). (c-d) Single cell transcriptional screening of multiple patient samples consistently identifies the hASC subpopulation following k-means clustering (k=2). Gene expression presented as fold change from median (yellow - high expression, 32-fold above median to blue - low expression, 32-fold below median; gray - no expression). (e) Creation of an assay list screening for gene expression related to tissue regeneration, cell stemness, survival and proliferation, in addition to selected surface markers. (f) K-means clustering (k=2) of hASCs with the functionalized gene list re-identifies the cell subpopulation, which displays enhanced expression of multiple genes related to tissue regeneration.

Supplementary Figure 3:

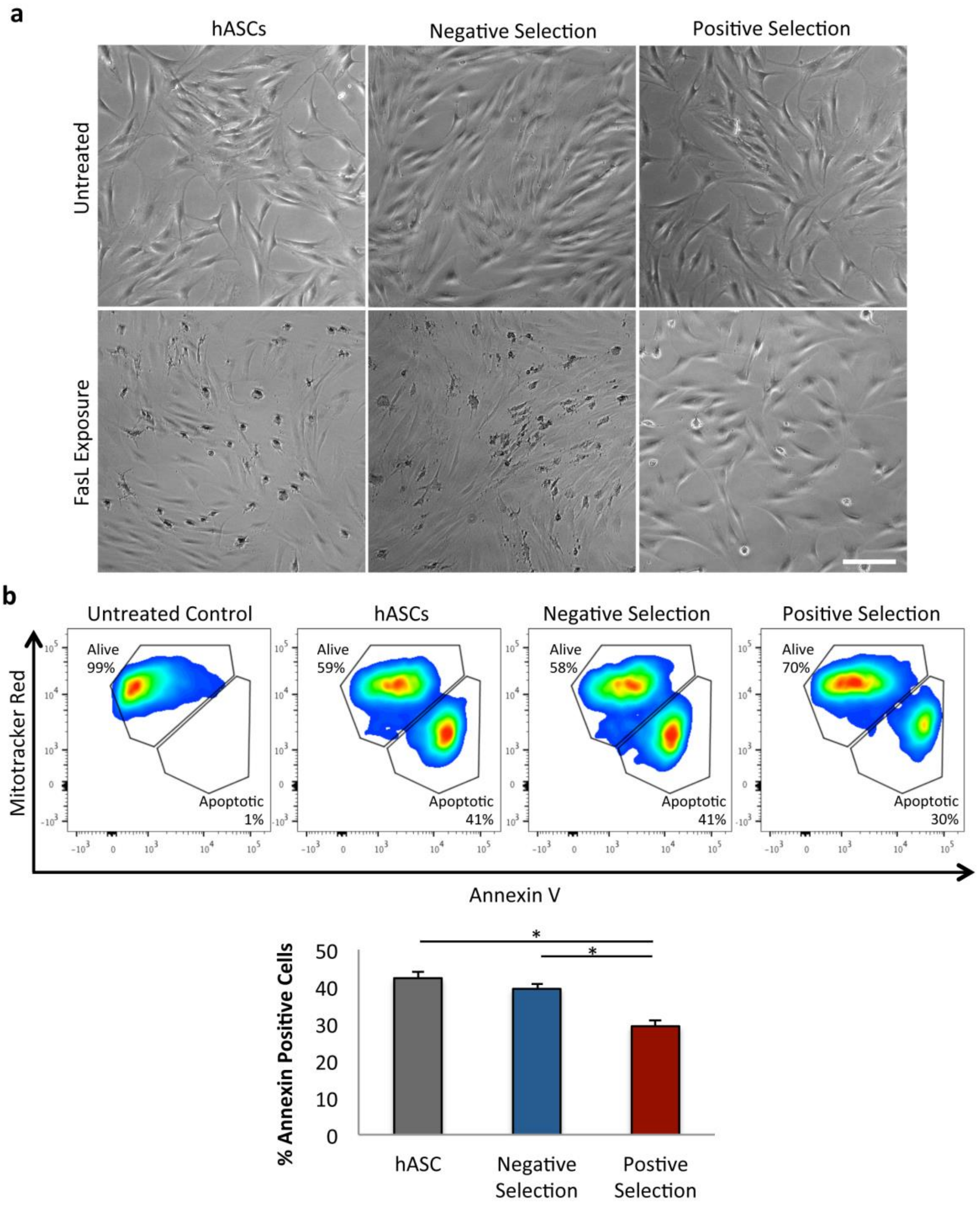

Supplementary Figure 3: Prospective hASC subpopulation enrichment enhances cell survival. (a) Light micrograph images of cells following exposure to an apoptotic stimulus (FasL; as seen in **Fig. 2a**) reveals enhanced survival in positively selected cells. (b) Analysis of downstream apoptotic signaling (Annexin V) following exposure to FasL confirms the survival benefit of hASC positive subpopulation selection. * indicates $p \leq 0.05$ via Student’s t-test. Error bars represent SEM. Scale bar = 50μm.

Supplementary Figure 4:

a

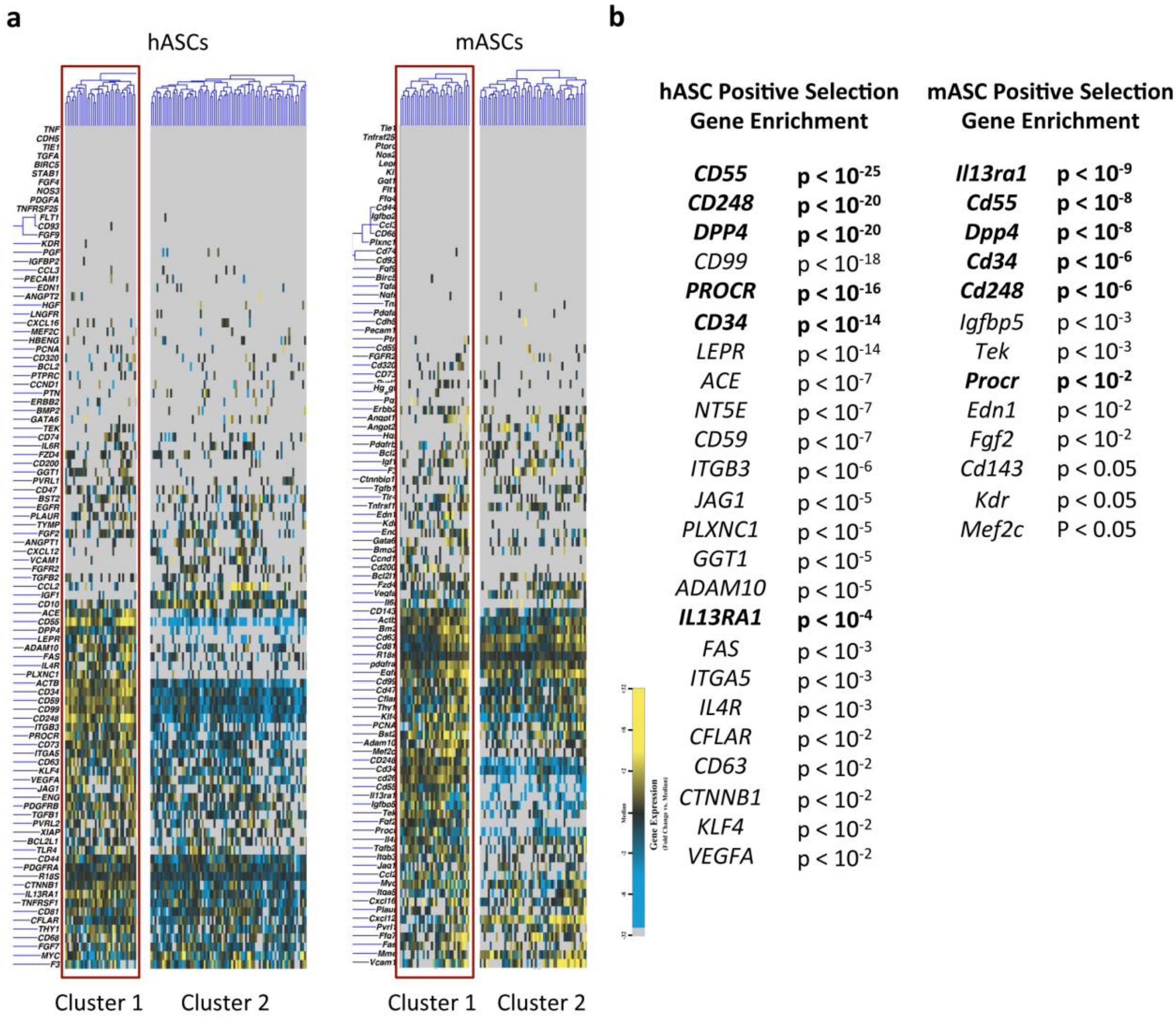

b

hASC Positive Selection Gene Enrichment	
CD55	$\mathbf{p < 10^{-25}}$
CD248	$\mathbf{p < 10^{-20}}$
DPP4	$\mathbf{p < 10^{-20}}$
CD99	$p < 10^{-18}$
PROCR	$\mathbf{p < 10^{-16}}$
CD34	$\mathbf{p < 10^{-14}}$
LEPR	$p < 10^{-14}$
ACE	$p < 10^{-7}$
NT5E	$p < 10^{-7}$
CD59	$p < 10^{-7}$
ITGB3	$p < 10^{-6}$
JAG1	$p < 10^{-5}$
PLXNC1	$p < 10^{-5}$
GGT1	$p < 10^{-5}$
ADAM10	$p < 10^{-5}$
IL13RA1	$\mathbf{p < 10^{-4}}$
FAS	$p < 10^{-3}$
ITGA5	$p < 10^{-3}$
IL4R	$p < 10^{-3}$
CFLAR	$p < 10^{-2}$
CD63	$p < 10^{-2}$
CTNNB1	$p < 10^{-2}$
KLF4	$p < 10^{-2}$
VEGFA	$p < 10^{-2}$

mASC Positive Selection Gene Enrichment	
Il13ra1	$\mathbf{p < 10^{-9}}$
Cd55	$\mathbf{p < 10^{-8}}$
Dpp4	$\mathbf{p < 10^{-8}}$
Cd34	$\mathbf{p < 10^{-6}}$
Cd248	$\mathbf{p < 10^{-6}}$
Igfbp5	$p < 10^{-3}$
Tek	$p < 10^{-3}$
Procr	$\mathbf{p < 10^{-2}}$
Edn1	$p < 10^{-2}$
Fgf2	$p < 10^{-2}$
Cd143	$p < 0.05$
Kdr	$p < 0.05$
Mef2c	$P < 0.05$

c

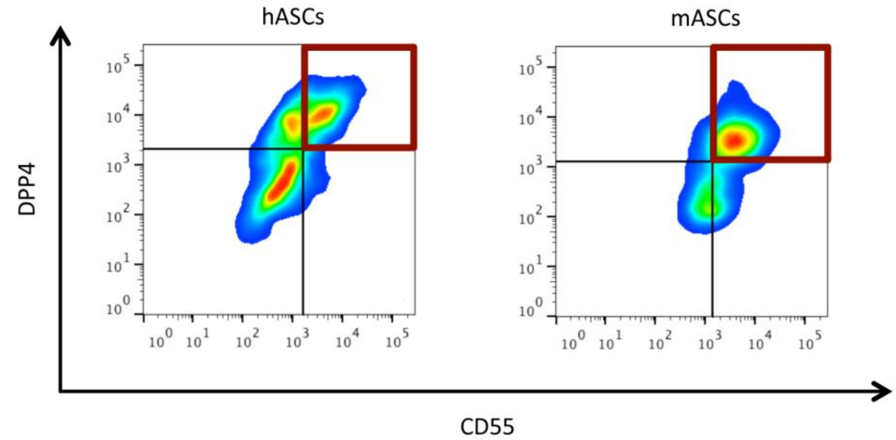

Supplementary Figure 4: The transcriptionally identified ASC subpopulation is present in both humans and mice. (a) Single cell transcriptional analysis for human and murine ASCs reveals the presence of a similar cell subpopulation. (b) Kolmogorov-Smirnov analysis of positively selected hASCs and mASCs reveals similar gene distribution profiles across species (list of non-control hASC and mASC genes positively regulated following subpopulation enrichment, with common genes bolded). (c) Flow cytometic confirmation of cross-species presence of the ASC subpopulation, as defined by the surface markers DPP4 and CD55.

Supplementary Figure 5:

a

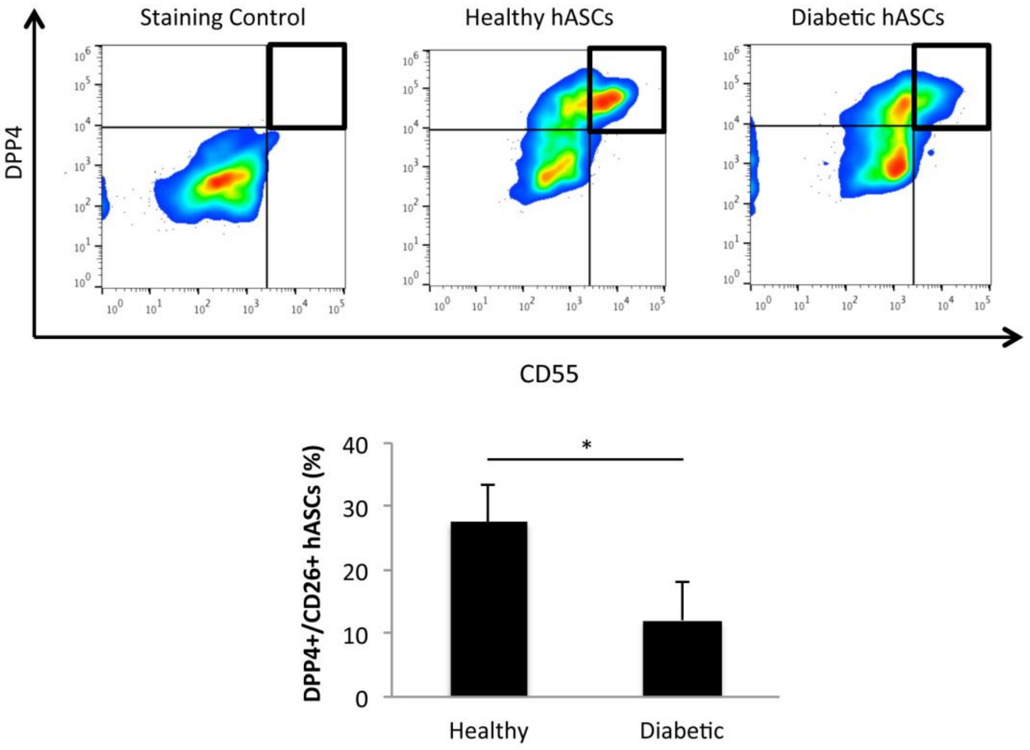

b

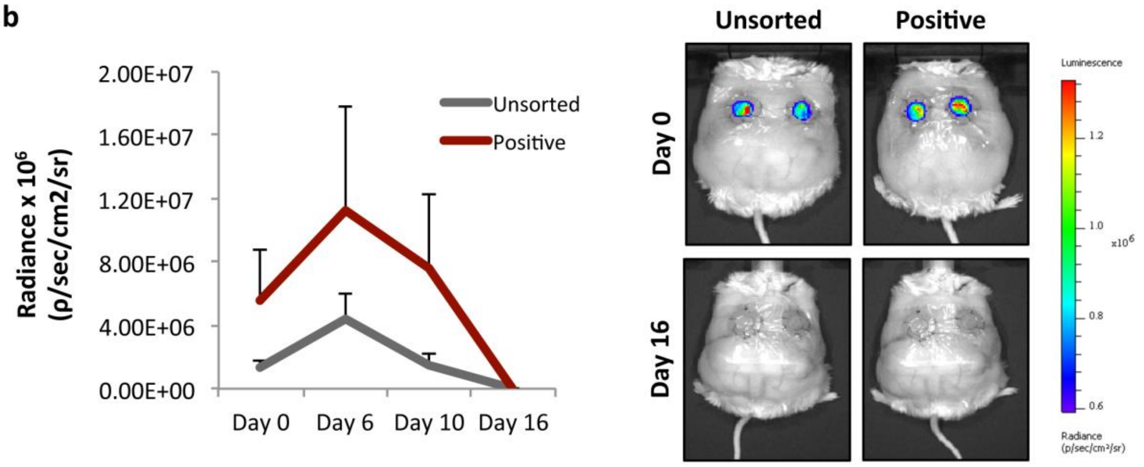

Supplementary Figure 5: Effect of diabetes on the transcriptionally identified hASC subpopulation and *in vivo* cell dynamics following application of enriched hASCs. (a) The transcriptionally identified hASC subpopulation is significantly depleted in patients with diabetes as compared to healthy, non-diabetic controls (bar graph represents total number of DPP4+/CD55+ ASCs as a fraction of total ASCs within the SVF). (b) hASCs persist for up to 16 days *in vivo* (assessed via IVIS tracking), with trends toward improved early engraftment/survival of enriched versus unsorted hASCs. * indicates $p \leq 0.05$ via Student's t-test. Error bars represent SEM.

Supplementary Figure 6:

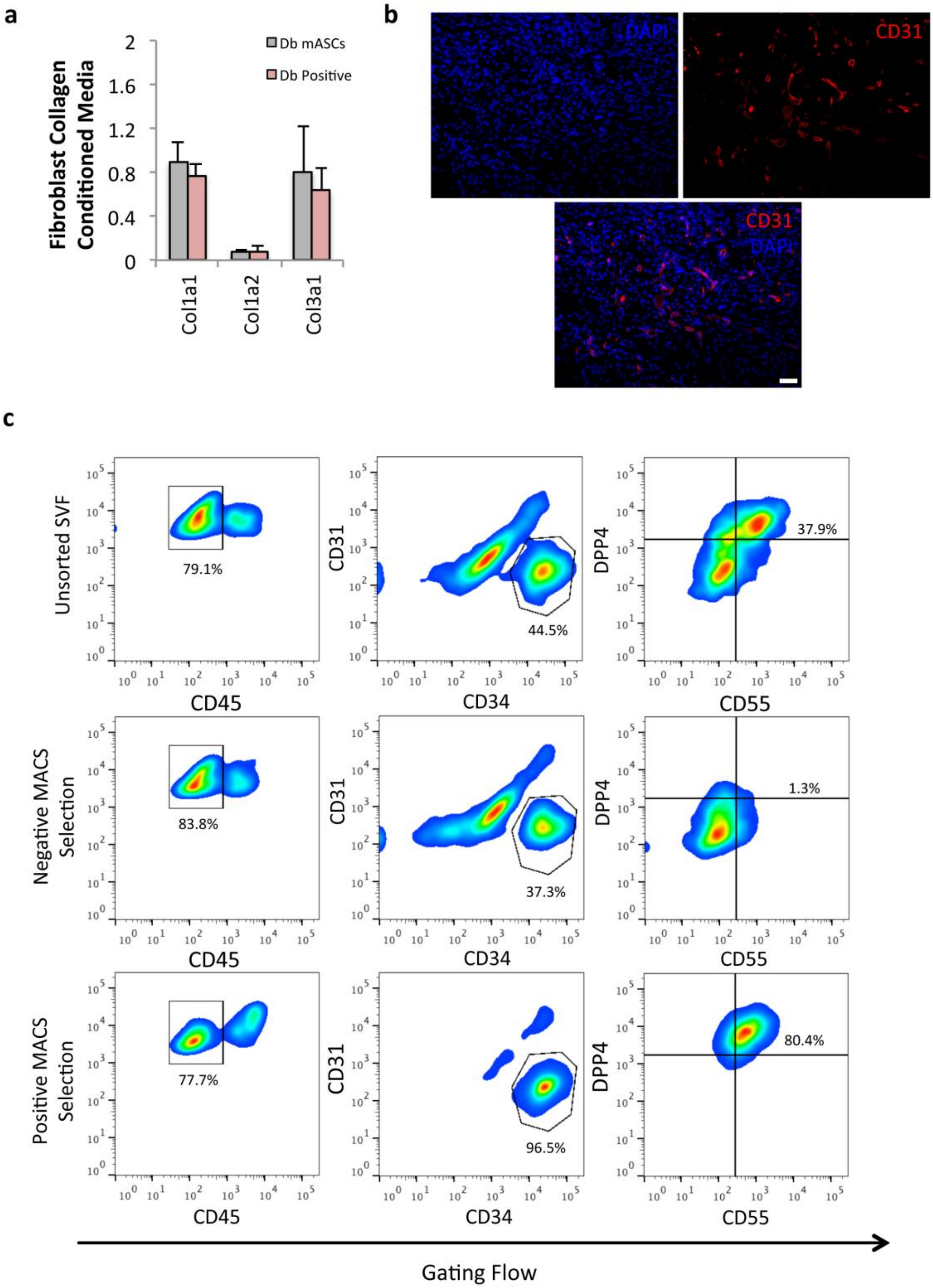

Supplementary Figure 6: Need for allogenic cell sourcing, treated wound vascularity, and demonstration of rapid subpopulation enrichment of hASCs via magnetic assisted cell sorting (MACS). (a) Unlike healthy ASCs, enrichment of diabetic mASCs failed to improved secondary fibroblast collagen production. (b) Diabetic wounds treated with enriched mASCs demonstrate robust vascularity immediately after closure via immunohistochemical staining for the endothelial cell marker CD31. Representative images; scale bar = 100μm. (c) Flow cytometic analysis of unsorted and subpopulation enriched and depleted hASCs via dual-antibody MACS confirms the ability to efficiently and rapidly isolate the identified hASC subpopulation for potential clinical application (%'s reported represent fraction of cells from the parent gate). Error bars represent SEM.

Supplementary Figure 7:

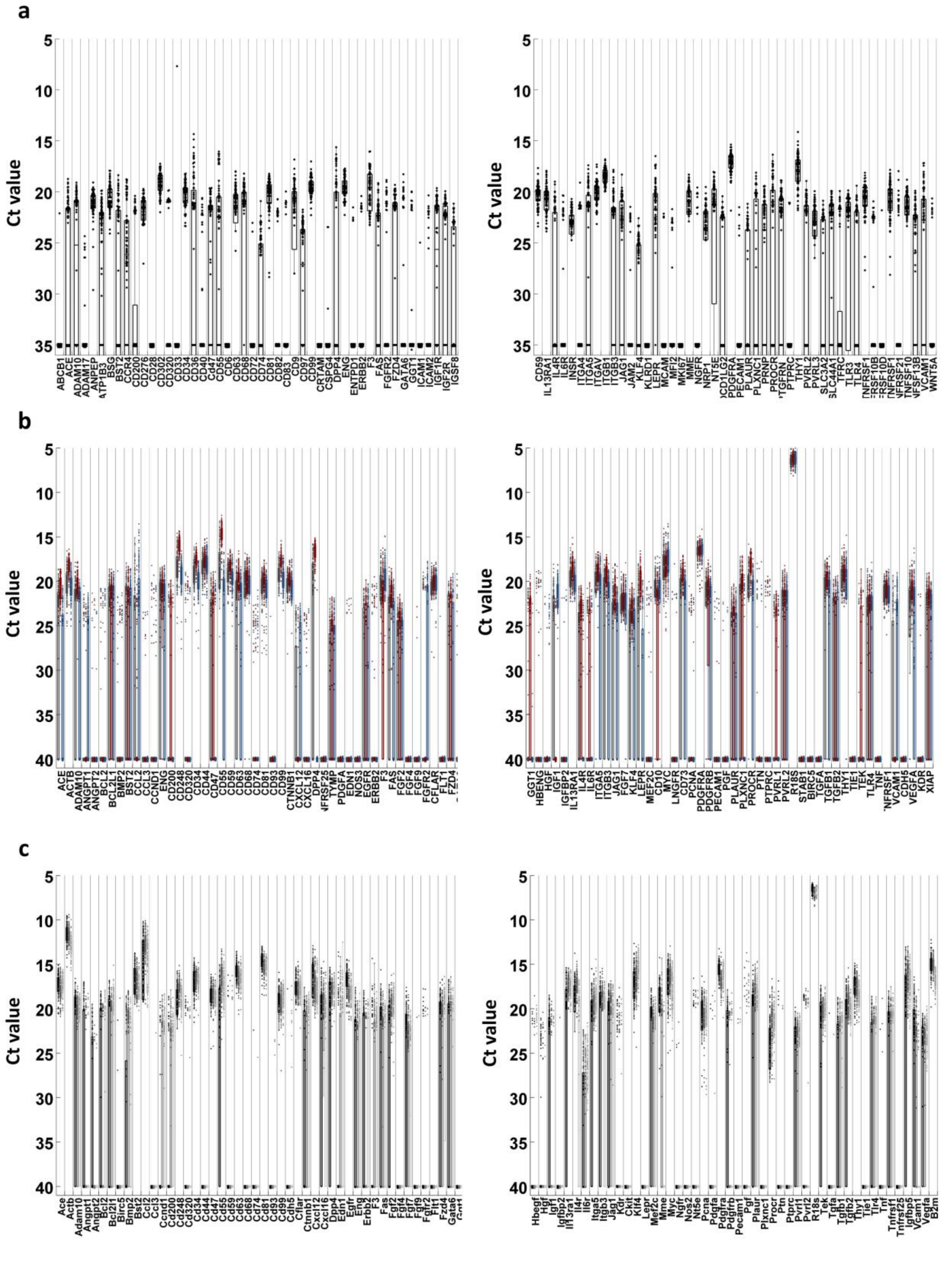

Supplementary Figure 7: Whisker plots. (a) Whisker plots presenting raw qPCR cycle threshold values for each gene across all cells for the initial single-cell gene distribution subpopulation analysis of hASCs. Informed by the n= 386 surface marker screen, high copy number, differentially expressed surface markers (>5 and <95% cell expression, with a cycle threshold [Ct] < 21) were preferentially included to distinguish biologically important cell subsets, in addition to selected control and cell-cycle related probes (full gene list available in **Supplementary Table 2**). Individual dots represent single gene/cell qPCR reactions, with increased cycle threshold values corresponding to decreased mRNA content. Cycle threshold values of 40 represent failed amplifications. (b) Whisker plots for functionalized single-cell gene distribution analyses of unsorted and enriched hASCs (gray = hASCs, red = positively selected cells, and blue = negatively selected cells). Genes specifically relating to tissue regeneration, cell stemness, survival, and proliferation were chosen, in addition to selected control and surface marker related probes (full gene list available in **Supplementary Table 3**). (c) Whisker plots for single-cell gene distribution analyses of mASCs (black = healthy mASCs, dark gray = aged mASCs, and light gray = diabetic mASCs). Genes specifically relating to tissue regeneration, cell stemness, survival, and proliferation were chosen, in addition to selected control and surface marker related probes (full gene list available in **Supplementary Table 4**).

Supplementary Table 1:

Differentially Distributed Subpopulation Genes	
CD55	$p < 10^{-8}$
PROCR	$p < 10^{-6}$
ACE	$p < 10^{-4}$
DPP4	$p < 10^{-4}$
ITGB3	$p < 10^{-3}$
IL4R	$p < 10^{-2}$
CD34	$p < 10^{-2}$
CD99	$p < 10^{-2}$
CD73	$p < 0.05$
PTGFRN	$p < 0.05$
GGT1	$p < 0.05$

Significance determined via Kolmogorov-Smirnov analysis.

Supplementary Table 2:

Gene list for initial hASC single-cell analysis

Assay ID	Gene Name	Assay ID	Gene Name
Hs00184500_m1	*ABCB1*	Hs00364603_m1	*IGSF8*
Hs00174179_m1	*ACE*	Hs00609817_m1	*IL13RA1*
Hs00153853_m1	*ADAM10*	Hs00166237_m1	*IL4R*
Hs01041915_m1	*ADAM17*	Hs01075667_m1	*IL6R*
Hs00174265_m1	*ANPEP*	Hs00961554_m1	*INSR*
Hs00740857_mH	*ATP1B3*	Hs00168433_m1	*ITGA4*
Hs00936295_m1	*BSG*	Hs01547673_m1	*ITGA5*
Hs00171632_m1	*BST2*	Hs00233808_m1	*ITGAV*
Hs99999919_m1	*CCR4*	Hs01127543_m1	*ITGB1*
Hs01033303_m1	*CD200*	Hs01001469_m1	*ITGB3*
Hs00987207_m1	*CD276*	Hs01070036_m1	*JAG1*
Hs00174796_m1	*CD28*	Hs01022013_m1	*JAM2*
Hs00994886_m1	*CD302*	Hs00358836_m1	*KLF4*
Hs00213164_m1	*CD320*	Hs00233844_m1	*KLRD1*
Hs00233544_m1	*CD33*	Hs00174497_m1	*LEPR*
Hs02576480_m1	*CD34*	Hs00174838_m1	*MCAM*
Hs00169627_m1	*CD36*	Hs00195551_m1	*MFI2*
Hs00374176_m1	*CD40*	Hs01032443_m1	*MKI67*
Hs00179953_m1	*CD47*	Hs00153510_m1	*MME*
Hs00892618_m1	*CD55*	Hs00609976_m1	*NGFR*
Hs00174141_m1	*CD59*	Hs00826128_m1	*NRP1*
Hs00198752_m1	*CD6*	Hs01573922_m1	*NT5E*
Hs00156390_m1	*CD63*	Hs00228839_m1	*PDCD1LG2*
Hs00154355_m1	*CD68*	Hs00998018_m1	*PDGFRA*
Hs00233564_m1	*CD72*	Hs01065282_m1	*PECAM1*
Hs00269961_m1	*CD74*	Hs00182181_m1	*PLAUR*
Hs00174717_m1	*CD81*	Hs00955621_m1	*PLXNC1*
Hs01017982_m1	*CD82*	Hs00175591_m1	*PRNP*
Hs00188486_m1	*CD83*	Hs00941182_m1	*PROCR*
Hs00233521_m1	*CD9*	Hs01385989_m1	*PTGFRN*
Hs00173542_m1	*CD97*	Hs00898488_m1	*PTPRC*
Hs00365982_m1	*CD99*	Hs01071562_m1	*PVRL2*
Hs00219699_m1	*CRTAM*	Hs00210043_m1	*PVRL3*
Hs00426981_m1	*CSPG4*	Hs00374243_m1	*SLC3A2*
Hs00175210_m1	*DPP4*	Hs00223114_m1	*SLC44A1*
Hs00923996_m1	*ENG*	Hs00951083_m1	*TFRC*
Hs00969559_m1	*ENTPD1*	Hs00174816_m1	*THY1*
Hs01001580_m1	*ERBB2*	Hs01551078_m1	*TLR3*
Hs01076029_m1	*F3*	Hs00152939_m1	*TLR4*
Hs00531110_m1	*FAS*	Hs01042313_m1	*TNFRSF1*
Hs01552926_m1	*FGFR2*	Hs00366278_m1	*TNFRSF10B*
Hs00201853_m1	*FZD4*	Hs00388742_m1	*TNFRSF10D*
Hs00934682_m1	*GATA6*	Hs01042313_m1	*TNFRSF1A*
Hs00980756_m1	*GGT1*	Hs00205419_m1	*TNFRSF21*
Hs00164932_m1	*ICAM1*	Hs00921974_m1	*TNFSF10*
Hs00609563_m1	*ICAM2*	Hs00198106_m1	*TNFSF13B*
Hs00609566_m1	*IGF1R*	Hs01003372_m1	*VCAM1*
Hs00974474_m1	*IGF2R*	Hs00998537_m1	*WNT5A*

Supplementary Table 3:

Gene list for functionalized single-cell analyses of unsorted and enriched hASCs

Assay ID	Gene Name
Hs00174179_m1	*ACE*
Hs99999903_m1	*ACTB*
Hs00153853_m1	*ADAM10*
Hs00375822_m1	*ANGPT1*
Hs01048042_m1	*ANGPT2*
Hs99999018_m1	*BCL2*
Hs00236329_m1	*BCL2L1*
Hs00153353_m1	*BIRC5*
Hs00154192_m1	*BMP2*
Hs00171632_m1	*BST2*
Hs00234140_m1	*CCL2*
Hs00234142_m1	*CCL3*
Hs00765553_m1	*CCND1*
Hs01033303_m1	*CD200*
Hs00535586_s1	*CD248*
Hs00213164_m1	*CD320*
Hs02576480_m1	*CD34*
Hs01075861_m1	*CD44*
Hs00179953_m1	*CD47*
Hs00892618_m1	*CD55*
Hs00174141_m1	*CD59*
Hs00156390_m1	*CD63*
Hs00154355_m1	*CD68*
Hs00269961_m1	*CD74*
Hs00174717_m1	*CD81*
Hs00362607_m1	*CD93*
Hs00365982_m1	*CD99*
Hs00174344_m1	*CDH5*
Hs00153439_m1	*CFLAR*
Hs00355049_m1	*CTNNB1*
Hs00930455_m1	*CXCL12*
Hs00222859_m1	*CXCL16*
Hs00175210_m1	*DPP4*
Hs00174961_m1	*EDN1*
Hs01076078_m1	*EGFR*
Hs00923996_m1	*ENG*
Hs01001580_m1	*ERBB2*
Hs01076029_m1	*F3*
Hs00531110_m1	*FAS*
Hs00266645_m1	*FGF2*
Hs00999691_m1	*FGF4*
Hs00940253_m1	*FGF7*
Hs00181829_m1	*FGF9*
Hs01552926_m1	*FGFR2*
Hs01052961_m1	*FLT1*
Hs00201853_m1	*FZD4*
Hs00934682_m1	*GATA6*
Hs00980756_m1	*GGT1*

Assay ID	Gene Name
Hs00181813_m1	*HB-EGF*
Hs00300159_m1	*HGF*
Hs01547656_m1	*IGF1*
Hs01040719_m1	*IGFBP2*
Hs00609817_m1	*IL13RA1*
Hs00166237_m1	*IL4R*
Hs01075667_m1	*IL6R*
Hs01547673_m1	*ITGA5*
Hs01001469_m1	*ITGB3*
Hs01070036_m1	*JAG1*
Hs00176676_m1	*KDR*
Hs00358836_m1	*KLF4*
Hs00174497_m1	*LEPR*
Hs01554597_m1	*MEF2C*
Hs00153510_m1	*MME*
Hs00905030_m1	*MYC*
Hs00609976_m1	*NGFR*
hs01574659_m1	*NOS3*
Hs01573922_m1	*NT5E*
Hs00696862_m1	*PCNA*
Hs00964426_m1	*PDGFA*
Hs00998018_m1	*PDGFRA*
Hs01019580_m1	*PDGFRB*
Hs00169777_m1	*PECAM1*
Hs00182176_m1	*PGF*
Hs00182181_m1	*PLAUR*
Hs00955621_m1	*PLXNC1*
Hs00941182_m1	*PROCR*
Hs00383235_m1	*PTN*
Hs00898488_m1	*PTPRC*
Hs00161050_m1	*PVRL1*
Hs01071562_m1	*PVRL2*
Hs99999901_s1	*R18S*
Hs01109068_m1	*STAB1*
Hs00945146_m1	*TEK*
Hs00608187_m1	*TGFA*
Hs00998133_m1	*TGFB1*
Hs00234244_m1	*TGFB2*
Hs00174816_m1	*THY1*
Hs00178500_m1	*TIE1*
Hs00152939_m1	*TLR4*
Hs99999043_m1	*TNF*
Hs01042313_m1	*TNFRSF1*
Hs00237054_m1	*TNFRSF25*
Hs00157317_m1	*TYMP*
Hs00174239_m1	*VCAM1*
Hs00900055_m1	*VEGFA*
Hs00236913_m1	*XIAP*

Supplementary Table 4:

Gene list for single-cell gene distribution analyses of mASCs

Assay ID	Gene Name
Mm00802048_m1	*Ace*
Mm00607939_s1	*Actb*
Mm00545742_m1	*Adam10*
Mm00456503_m1	*Angpt1*
Mm00545822_m1	*Angpt2*
Mm00437762_m1	*B2m*
Mm00477631_m1	*Bcl2*
Mm00437783_m1	*Bcl2l1*
Mm00599749_m1	*Birc5*
Mm01340178_m1	*Bmp2*
Mm01609165_g1	*Bst2*
Mm00441242_m1	*Ccl2*
mm00441259_g1	*Ccl3*
Mm00432359_m1	*Ccnd1*
Mm00487740_m1	*Cd200*
Mm00547485_s1	*Cd248*
Mm00507988_m1	*Cd320*
Mm00519283_m1	*Cd34*
Mm00681165_m1	*Cd44*
Mm00495005_m1	*Cd47*
Mm00438377_m1	*Cd55*
Mm00483149_m1	*Cd59*
Mm01966817_g1	*Cd63*
Mm03047340_m1	*Cd68*
Mm00658576_m1	*Cd74*
Mm00504869_m1	*Cd81*
Mm00440239_g1	*Cd93*
Mm04214669_u1	*Cd99*
Mm00486938_m1	*Cdh5*
Mm01255578_m1	*Cflar*
Mm00517812_m1	*Ctnnb1*
Mm00445552_m1	*Cxcl12*
Mm00469712_m1	*Cxcl16*
Mm00494538_m1	*Dpp4*
Mm00438656_m1	*Edn1*
Mm00433023_m1	*Egfr*
Mm00468256_m1	*Eng*
Mm00658541_m1	*Erbb2*
mm00438853_m1	*F3*
mm01204974_m1	*Fas*
Mm00433287_m1	*Ffg2*
Mm00438917_m1	*Ffg4*
Mm00433291_m1	*Ffg7*
Mm00442795_m1	*Fgf9*
Mm01269930_m1	*Fgfr2*
Mm00438980_m1	*Flt1*
Mm00433382_m1	*Fzd4*
Mm01235633_m1	*Gata6*

Assay ID	Gene Name
Mm00492322_m1	*Ggt1*
Mm00439306_m1	*Hg-egf*
Mm01135193_m1	*Hgf*
Mm00439560_m1	*Igf1*
Mm00492632_m1	*Igfbp2*
Mm00516037_m1	*Igfbp5*
Mm00446726_m1	*Il13ra1*
Mm01275139_m1	*Il4r*
Mm00439653_m1	*Il6r*
Mm00439797_m1	*Itga5*
Mm00443980_m1	*Itgb3*
Mm00496902_m1	*Jag1*
Mm01222421_m1	*Kdr*
Mm00445212_m1	*Kit*
Mm00516104_m1	*Klf4*
Mm00440181_m1	*Lepr*
Mm01340842_m1	*Mef2c*
Mm00485028_m1	*Mme*
Mm00487803_m1	*Myc*
Mm00446296_m1	*Ngfr*
Mm00440502_m1	*Nos2*
Mm00501910_m1	*Nt5e*
Mm00448100_g1	*Pcna*
Mm01205760_m1	*Pdgfa*
Mm00440701_m1	*Pdgfra*
Mm00435546_m1	*Pdgfrb*
Mm01242584_m1	*Pecam1*
Mm01302896_m1	*Pgf*
Mm00440911_m1	*Plaur*
Mm01236713_m1	*Plxnc1*
Mm00440992_m1	*Procr*
Mm01132688_m1	*Ptn*
Mm01292575_m1	*Ptprc*
Mm00445392_m1	*Pvrl1*
Mm00436144_m1	*Pvrl2*
Mm03928990_g1	*R18s*
Mm00443243_m1	*Tek*
Mm00446232_m1	*Tgfa*
Mm01178820_m1	*Tgfb1*
Mm00436955_m1	*Tgfb2*
Mm00493681_m1	*Thy1*
Mm00441786_m1	*Tie2*
Mm00445273_m1	*Tlr4*
Mm00443258_m1	*Tnf*
Mm00441875_m1	*Tnfrsf1*
Mm01263821_m1	*Tnfrsf25*
Mm01320970_m1	*Vcam1*
Mm01281447_m1	*Vegfa*
